# Supplementary material for: A fault-intrusion-tolerant system and deadline-aware algorithm for scheduling scientific workflow in the cloud
Source: PeerJ Comput Sci. 2021 Nov 2;7:e747. doi: 10.7717/peerj-cs.747 (PMC8576554; doi:10.7717/peerj-cs.747)
Supplement: Supplemental Information 6 [file peerj-cs-07-747-s006.zip › Myproject1.1/docs/org/cloudbus/cloudsim/class-use/CloudletSchedulerTimeShared.html]

Uses of Class org.cloudbus.cloudsim.CloudletSchedulerTimeShared


JavaScript is disabled on your browser.


- Overview
- Package
- Class
- Use
- Tree
- Deprecated
- Index
- Help

- Prev
- Next

- Frames
- No Frames

- All Classes

## Uses of Class org.cloudbus.cloudsim.CloudletSchedulerTimeShared

- Packages that use CloudletSchedulerTimeShared

  | Package | Description |
  | --- | --- |
  |  |  |
  | --- | --- |
  | org.cloudbus.cloudsim |  |
- - ### Uses of CloudletSchedulerTimeShared in org.cloudbus.cloudsim

    Subclasses of CloudletSchedulerTimeShared in org.cloudbus.cloudsim

    | Modifier and Type | Class and Description |
    | --- | --- |
    |  |  |
    | --- | --- |
    | `class` | `CloudletSchedulerDynamicWorkload` CloudletSchedulerDynamicWorkload implements a policy of scheduling performed by a virtual machine assuming that there is just one cloudlet which is working as an online service. |

- Overview
- Package
- Class
- Use
- Tree
- Deprecated
- Index
- Help

- Prev
- Next

- Frames
- No Frames

- All Classes
